# Time to consider oesophageal atresia as a life-long disease

**DOI:** 10.1097/JS9.0000000000001167

**Published:** 2024-02-19

**Authors:** Mélanie Leroy, Madeleine Aumar, David Seguy, Florent Vandamme, Anke Widenmann, Rony Sfeir, Frédéric Gottrand

**Affiliations:** a University Lille, Reference Center for Congenital esophageal Anomalies, Lille, France; b Esophageal Atresia Global Support Groups e.V., Sommerrainstrasse, Stuttgart, Germany

Esophageal atresia (EA) is a rare congenital malformation comprising discontinuity of the esophagus with or without persistent communication with the trachea. EA was initially described in 1697 by Thomas Gibson. Before the first successful anastomosis in 1950, no infants born with EA did survive^1^. Before the 1980s, early complications and death directly related to EA occurred frequently, leading to a very few adults survivors^2^. However, given the last 30 years improvements in neonatal surgery, intensive care, and anesthesia, more than 92% of infants born with EA in developed countries now survive the neonatal period^3^. Structured follow-up, early nutritional support, proton pump inhibitors, and optimization of medical respiratory treatment are also responsible of increasing life expectancy. Therefore, long-term outcomes – including gastrointestinal, nutritional, and respiratory – have become an emerging concern in this population. As a consequence, the frequency of severe complications is now increasing. In a recent review by Aumar *et al*.^4^, the authors reported 13 cases of esophageal cancer (nine epidermoids and four adenocarcinomas) in patients born with EA, with a mean age of incidence of 42 years old (20–60 years). Others more frequent complications have also been described as GERD, which prevalence in adulthood has been estimated at 42.4% (95% CI: 33.2–52.1) in a recent systematic review from Brooks *et al*.^5^ based on 16 studies including 830 patients. The prevalence of ‘respiratory sequelae’ have also been estimated at 33.3% (95% CI: 10.1–69), based on the same review of six studies including 115 patients^5^. Undernutrition has also been a high prevalence in adult [estimated prevalence of 19.6% (95% CI: 11.6–31.1)]^5^. In 2008, our team implemented the first population-based registry of live births of infants with EA in the world. This registry now includes more than 2200 children born with EA in France, and the number of new cases is increasing by around 170 per year. This corresponds to an incidence of 1.8–2.0 cases per 10 000 live births. EA has an early mortality rate of 7%^6,7^, which decreases after the first year of life. We recently found a low mortality of 0.8% in a nested cohort of 360 patients on the same registry after a 6-year follow-up (*unpublished data*).

Based on this information, we modeled an age pyramid of the number of EA patients in France each year from 2020 to 2120 (Supplementary Video 1, Supplemental Digital Content 1, http://links.lww.com/JS9/B997). This model assumes a 2/10 000 incidence, mortality rates from birth to 1 year of age of 100% for patients born before 1950, 50% for those born between 1950 and 1980, and 5% for those born after 1980, and the expected mortality rate in the French population after 1 year of age.

**This projection reveals a large increase in the number of adult patients in the coming decades, which should lead to a population of more than 7000 adults with EA living in France in 2030 (and more than 40 000 in Europe). This would include 539 patients older than 60 years, and the first centenarian operated at birth for EA is expected in 2050. A similar increase may be anticipated in every high-income country, which will make the management of adults with EA a global concern.

Defined as a pediatric surgical disease for decades, EA should now be considered a life-long disease given that two-thirds of patients are now adults. As they reach the edge of the pediatric scope, patients experience other major changes in their life and their family, which emphasizes the importance of a smooth transition to adult specialists. All actors involved in the care and support of these patients should realize the impact of this shift in the population. The health system needs to acknowledge these figures and adapt by supporting the implementation of health-care transition programs. Adult specialists, especially gastroenterologists and pulmonologists, will increasingly have to care for patients with EA who have a long medical history and frequently a high level of knowledge about their disease. Considering the risk of uneven care management across centers, the transition should be multidisciplinary, involve patients as well as their parents, and be performed in specialized referral centers for best practice. Support groups should also extend their activities to include adult patients, which will have benefits by supporting adult patients who can then share their experiences.

Every year, thousands of patients with EA will reach adulthood worldwide regardless of whether the health-care transition is ready. Warning the medical community, training specialists to address these patients’ specific needs, and preparing for the transition are urgently needed to avoid losses in follow-up and late diagnosis of complications in adulthood, and to ensure optimal quality of life for adults with EA.

## Ethical approval

Not applicable.

## Consent

Not applicable.

## Sources of funding

Not applicable.

## Author contribution

M.L.: drafted the manuscript; F.G.: did the study concept. All authors revised the manuscript for important intellectual content.

## Conflicts of interest disclosure

Not applicable.

## Research registration unique identifying number (UIN)

Not applicable.

## Guarantor

Pr Frédéric Gottrand.

## Data availability statement

Data are available upon reasonable request to the corresponding author.

## Provenance and peer review

Not commissioned.

## Supplementary Material
